# Relationship between Tolerance and Persistence Mechanisms in Acinetobacter baumannii Strains with AbkAB Toxin-Antitoxin System

**DOI:** 10.1128/AAC.00250-18

**Published:** 2018-04-26

**Authors:** Laura Fernández-García, Felipe Fernandez-Cuenca, Lucía Blasco, Rafael López-Rojas, Anton Ambroa, María Lopez, Álvaro Pascual, Germán Bou, María Tomás

**Affiliations:** a Servicio de Microbiología, Complejo Hospitalario Universitario A Coruña (CHUAC)—Instituto de Investigación Biomédica (INIBIC), A Coruña, Spain; b Unidad de Enfermedades Infecciosas y Microbiología Clínica, Hospital Universitario Virgen Macarena, Seville, Spain; c Departamento de Microbiología. Universidad de Sevilla, Seville, Spain; d Instituto de Biomedicina de Sevilla (IBIS), Seville, Spain

**Keywords:** tolerance, persistence, chlorhexidine, imipenem, Acinetobacter, toxin-antitoxin

## Abstract

The molecular mechanisms of tolerance and persistence associated with several compounds in Acinetobacter baumannii clinical isolates are unknown. Using transcriptomic and phenotypic studies, we found a link between mechanisms of bacterial tolerance to chlorhexidine and the development of persistence in the presence of imipenem in an A. baumannii strain belonging to clinical clone ST-2 (OXA-24 β-lactamase and AbkAB toxin-antitoxin [TA] system carried in a plasmid). Interestingly, the strain A. baumannii ATCC 17978 (AbkAB TA system from plasmid) showed persistence in the presence of imipenem and chlorhexidine.

## TEXT

The importance of preventing the development of tolerance and/or persistence has recently been highlighted as a new strategy for delaying the emergence of resistance (1–4). In this context, it is essential to distinguish between bacterial resistance, tolerance, and persistence (5). Resistance refers to the ability of bacterial populations to grow at the same rate in the presence of antibiotic-induced or environmental stress. Tolerance is the ability of a bacterial population to grow slowly in response to stress. Finally, persistence is the latent state of a bacterial subpopulation, which is activated under certain conditions (5).

Several bacterial tolerance mechanisms develop during stress and antibiotic exposure (6). These mechanisms include (p)ppGpp signaling accumulation, reactive oxygen species (ROS) and SOS responses, bacterial communication (quorum sensing), efflux pumps, and energy metabolism (6).

Carbapenem-resistant Acinetobacter baumannii (CRAb) is currently a major source of nosocomial infections and is considered a highly successful human pathogen (7). Among the different mechanisms associated with carbapenem resistance in A. baumannii, the production of acquired carbapenem-hydrolyzing class D β-lactamases (CHDLs) and class B metallo-β-lactamases (MBLs) has been widely studied (8). On the other hand, the main mechanisms of development of persister cells in the presence of antibiotics (such as imipenem [IMP]) involve toxin-antitoxin (TA) modules (6, 9).

Studies about molecular mechanisms of tolerance and persistence from A. baumannii strains in response to several compounds are scarce. In this study, we used transcriptomic and phenotypic assays to analyze the tolerance and persistence mechanisms of A. baumannii isolates in response to chlorhexidine and imipenem (resistance and susceptibility to carbapenems).

In a previous work of the REIPI-GEIH Ab-2010 project (10), we worked with the A. baumannii clinical strains Ab-2_clon_2010 (belonging to clone ST-2) and Ab-2_clon_2010-CHLX, which showed the absence of an increase of MICs to antibiotics after exposure to subinhibitory concentrations of chlorhexidine digluconate (CHLX) (0.25× MIC) during 4 weeks (see Table S1 in the supplemental material). The genome of this Ab-2_clon_2010 strain, together with 17 other clinical strains from this ST-2 clone, were sequenced by Lopez et al. (11) in the Umbrella GenBank BioProject number PRJNA422585. All strains from this ST-2 clone belonged to the REIPI-GEIH Ab-2010 project and had a plasmid with the *bla*OXA_24/40_ β-lactamase gene (conferring resistance to carbapenems), as well as the *abkAB* genes from a toxin-antitoxin system (12). RNA assays by transcriptomics had a number of reads assigned to the different genes and were analyzed using the EdgeR and DESeq2 packages and reverse transcription PCR (RT-PCR) techniques using UPLs Probe (see Table S3 in the supplemental material; Roche, Germany) of both clinical isolates (DNase-treated RNA of Ab-2_clon_2010 and Ab-2_clon_2010-CHLX) (GenBank BioProject number PRJNA433173 and GEO series number GSE110207), the results of which are shown in Table S2 and Fig. S1 and S2 in the supplemental material.

The results showed the activation of tolerance molecular mechanisms (known as “tolerome”) in response to chlorhexidine in strain Ab-2_clon_2010-CHLX (Table 1). In relation with the tolerome, in the strain Ab-2_clon_2010-CHLX, we observed overexpression (1.5- to 6-fold change [FC]) of genes encoding the AdeABC, arsenite, and AceI chlorhexidine efflux pumps (10, 13–16). Some of these additional protective mechanisms, such as the production of efflux pumps, may also reduce the effective concentration of the antibiotic, which increases the MIC and results in a mixed phenotype of resistance and tolerance (5). We also observed an increase in the expression of genes involved in tetracycline and aminoglycoside resistance (FC, 3.4 to 6). The genes with the highest level of overexpression in this study were those carried by the AbATCC329 plasmid (PMMCU3p), such as OXA24/40 β-lactamase, DNA replication protein, and OriV (FC, 5.2 to 12) (12) (Table 1). Interestingly, the gene expression FCs of *abkA* (antitoxin gene) and *abkB* (toxin gene) from this plasmid were 0. 63 and 1.25, respectively. In addition, we observed the overexpression of genes associated with molecular mechanisms of bacterial tolerance (FC, 3.5 to 10), namely, the CsuA/BABCDE operon (17, 18), the CydAB operon (cytochrome *d* ubiquinol oxidase complex) (19, 20, 21), the taurine operon complex (taurine metabolism/electron carrier activity) (22, 23), and finally, regulatory genes involved in the quorum-sensing (QS) system, i.e., *abaR* and *abaI* (Table 1) (22–25).

**TABLE 1:** Mechanisms of bacterial tolerance to chlorhexidine in strain Ab-2_clon_2010-CHLX, revealed by transcriptomic studies^*a*^

GenBank^*b*^ protein accession no.	Gene expression fold change determined by:		Functional description	Defense mechanism (reference no.)	Tolerome type (reference no.)
	DESeq2	EdgeR			
ODA53993.1	6.933753475	6.982635042	AdeA protein	AdeABC system (RND-type) (10)	Transporter/efflux pump (5)
ODA53994.1	6.149907892	6.175526694	AdeB protein		
ODA53995.1	4.257153566	4.270842036	AdeC protein		
ODA55718.1	6.119321647	6.133494454	Tetracycline resistance protein	MFS system	
ODA54617.1	5.377292457	7.227172031	Arsenite efflux pump	ACR3 system (13)	
ODA56577.1	3.498098186	3.528728206	Aminoglycoside phosphotransferase	APT family	
ODA54814.1	3.605781331	3.649808635	Chlorexidine efflux pump	AceI system (16)	
ODA56167.1	5.265550668	7.054151044	MFS transporter	MFS system	
ODA53764.1	12.16763575	14.92175121	OXA 24/40 β-lactamase	AbATCC329p/pMMCU3	Plasmid (5)
ODA53763.1	8.975633873	11.30715263	DNA replication protein A		
ODA53762.1	5.273985066	5.329333593	RepB family plasmid replication initiator		
ODA54084.1	3.511019975	3.547062538	CsuA protein	CsuABCDE (17, 18)	Biofilm (14)
ODA54083.1	3.199749378	3.259685195	CsuB protein		
ODA54082.1	2.575094974	2.584527435	CsuC protein		
ODA54081.1	2.810613341	2.819199271	CsuD protein		
ODA54080.1	2.782552791	2.791313686	CsuE protein		
ODA53940.1	2.037734523	2.053934504	Cytochrome *b*	Cytochrome operon (19–21)	Stress oxidative (ROS) (21)
ODA57053.1	2.173049691	2.184371809	Cytochrome bd biosynthesis protein		
ODA56663.1	2.405101873	2.428897655	Sodium/proline symporter		
ODA56171.1	10.75708444	13.32903693	Cytochrome bd biosynthesis protein		
ODA56172.1	10.35093438	12.86380541	Cytochrome *d* ubiquinol oxidase subunit		
ODA54604.1	10.07652823	12.56398102	Taurine ABC transporter substrate-binding	Taurine transporter (22, 23)	Electron transport
ODA54605.1	9.758316312	12.21616998	Taurine transporter-binding subunit (TauB)		
ODA54606.1	8.966908008	11.30350134	Taurine ABC transporter permease (TauC)		
ODA54607.1	10.85324686	13.44475271	Taurine dioxygenase (TauD)		
ODA55153.1	−6.486154998	−6.530626555	Hypothetical protein	Replication	ppGpp network (28)^*c*^
ODA54592.1	0.932475218	0.929842277	DNA polymerase I		
ODA54625.1	0.931688577	0.929428817	DNA polymerase III subunit alpha		
ODA54730.1	−1.77207536	−1.816317901	Response regulator		
ODA55878.1	0.500078184	0.506140675	50S ribosomal protein L17		
ODA55763.1	0.438241011	0.436148678	RNA polymerase subunit omega		
ODA55654.1	0.582523178	0.580921263	50S ribosomal protein L7/L12		
ODA55933.1	−0.523115918	−0.531876133	ATP synthase subunit beta	ATP metabolism	Energy production (31, 32)^*c*^
ODA55935.1	−0.570647356	−0.579254835	ATP synthase subunit alpha		
ODA54585.1	0.422390483	0.418615357	Transcription termination factor rho		

a The relative expression (expressed as fold change [FC]) of *abaI* (3.05) and *abaR* (2.88) genes, determined by RT-PCR, indicated activation of the quorum-sensing system.

b Sanger sequencing of these genes from the Ab-2_clon_2010-CHLX strain, as well as of the regulatory genes *adeR* and *adeS*, did not show mutations with respect to the sequence of strain Ab-2_clon_2010.

c Genes that belonged to the ppGpp network and energy production categories showed downregulation (FC, <1).

We used the Kyoto Encyclopedia of Genes and Genomes (KEGG) tool to analyze those genes that were downregulated (FC, ≤0.5-fold) in Ab-2_clon_2010-CHLX. We studied two metabolic pathways. The first one was the ppGpp network (KEGG accession numbers ec00230 2.7.7.6 and ec00230 2.7.7.7) involving RNA polymerases, DNA polymerases, and finally, 50S ribosomal protein. The ppGpp network is mediated by a variety of RelA/SpoT homologue (RSH) proteins with a nucleotidyl transferase domain, with some displaying only synthetic or hydrolytic activities, and others displaying both (Rel) (26, 27). Accumulation of (p)ppGpp affects resource-consuming cell processes, such as replication, transcription, and translation. Furthermore, (p)ppGpp is thought to bind RNA polymerase and alter the transcriptional profile, decreasing the synthesis of translational machinery (such as rRNA and tRNA) and increasing transcription of the biosynthetic gene (28). Additionally, initiation of new rounds of replication is inhibited, and the cell cycle arrests until nutrient conditions improve (29). Translational GTPases involved in protein biosynthesis are also affected by ppGpp, with initiation factor 2 (IF2) being the main target (30). Although these proteins are scarcely known in A. baumannii, in this study we describe RelA-SpoT-homologous (RSH) proteins associated with these functions that show repression in Ab-2_clon_2010-CHLX isolate. The second metabolic pathway studied was that of oxidative phosphorylation (KEGG accession no. ec00190 3.6.3.14, ATP phosphohydrolases), in which the alpha/beta ATP synthase subunit and transcription termination factor rho were downregulated. Finally, energy production by ATP metabolism has been associated with the development of tolerant cells in Escherichia coli (31). Moreover, Wang et al., described how genes mapped in this pathway have an important role in the survival of clinical strains of Staphylococcus aureus (32).

**FIG 1:**
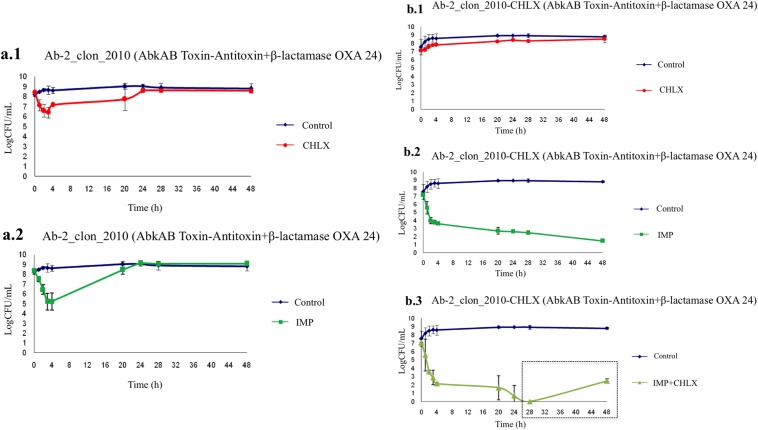
Time-kill curves in the presence of biocides (CHLX) and antibiotics (IMP) in Ab-2_clon_2010 (carbapenem-resistant) and Ab-2_clon_2010-CHLX isolates. Box in panel b.3, regrowth is due to putative reactivation of persister cells.

**FIG 2:**
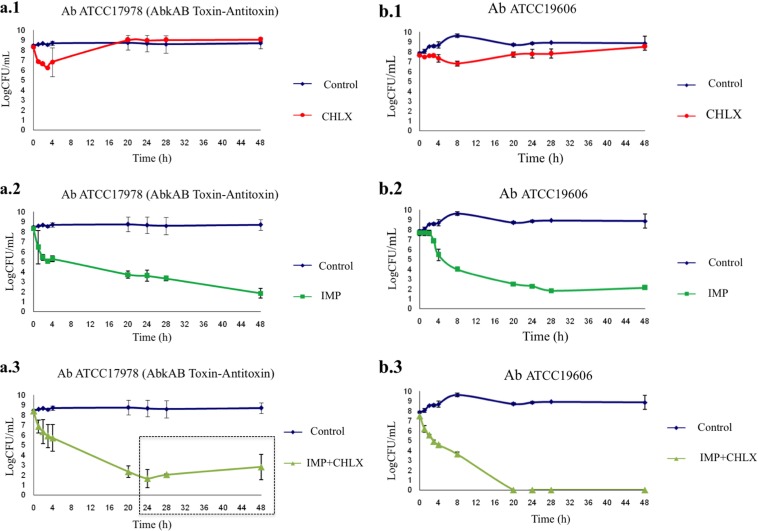
Time-kill curves in the presence of antibiotics (IMP) and biocides (CHLX) in susceptible A. baumannii ATCC strains. (a) A. baumannii strain ATCC 17978, which harbors the plasmid with the AbKA/AbkB toxin-antitoxin system (positive control); (b) A. baumannii ATCC 19606 strain without this AbKA/AbkB toxin-antitoxin system (negative control). Box in panel a.3, regrowth is due to putative reactivation of persister cells.

**FIG 3:**
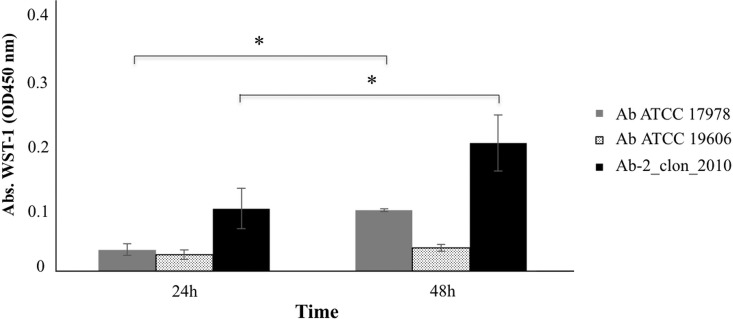
Enzymatic activity by colorimetric assay (WST-1-based) of the isolates A. baumannii ATCC 17978, A. baumannii ATCC 19606, and A. baumannii Ab-2_clon_2010-CHLX in the presence of IMP and CHLX. The *x* axis represents absorbance (optical density at 450 nm [OD_450_]), and the *y* axis represents time (h). *, *P* < 0.05 (Student's *t* test).

The time-kill curves for strains Ab-2_clon_2010 and Ab-2_clon_2010-CHLX were performed following the indications of Hofsteenge and colleagues (33) in low-nutrient Luria-Bertani broth (LN-LB; 2 g/liter tryptone, 1 g/liter yeast extract, and 5 g/liter NaCl) (13, 16). The cultures were incubated for 4 h to ensure logarithmic growth, and CHLX (0.25× MIC) and IMP (10× MIC) were then added alone or in combination to the cultures. We observed a lower growth rate of the Ab-2_clon_2010 strain in the presence of CHLX than in its absence (Fig. 1), as well as faster growth rate in the presence of IMP. Interestingly, the time-kill curves for isolate Ab-2_clon_2010-CHLX showed a massive killing in the presence of IMP (Fig. 1). The results of RT-PCR analysis confirmed a lower expression of OXA_24/40_ β-lactamase and *abkA* antitoxin genes (FC, 0.06 and 0.04, respectively) in Ab-2_clon_2010-CHLX, as well as overexpression of the *abkB* toxin gene (FC, 2.77) relative to that in Ab-2_clon_2010 (known as the persistome) (34–36). This toxin protein belongs to the AbkAB toxin-antitoxin module in the AbATCC329p/pMMCU3 plasmid (12). Mosqueda et al. located the AbkB/AbkA TA system (the so-called SplTA) in the most prevalent plasmids (GenBank KJ534568 and KJ534569) found in clinical isolates of A. baumannii (12, 37). Finally, we observed regrowth of persister cells in the Ab-2_clon_2010-CHLX isolate grown in the presence of IMP+CHLX for 28 h (Fig. 1). Moreover, we used two A. baumannii ATCC isolates as controls (both susceptible to carbapenems) whose complete genomes have been sequenced, A. baumannii strain ATCC 17978 (which harbors the AbkAB toxin-antitoxin system encoded by plasmid pAB2, GenBank number CP000523.1) and A. baumannii strain ATCC 19606 (which does not have this AbkAB toxin-antitoxin system). In Fig. 2, we observed that in the A. baumannii strain ATCC 17978, there was a reactivation of growth in the presence of IMP+CHLX for 28 h, in contrast to the lack of growth of the A. baumannii ATCC 19606 under the same conditions. These results of regrowth in the A. baumannii strain ATCC 17978 and in Ab-2_clon_2010-CHLX with IMP (10× MIC) and CHLX (0.25× MIC) at 48 h were confirmed by enzymatic analysis using the cell proliferation reagent WST-1 protocol (Roche, Germany) and calculating the serial dilutions of each culture (CFU/ml; Fig. 3).

In conclusion, this is the first study describing the important link between mechanisms of bacterial tolerance and persistence under chlorhexidine and imipenem pressure in a clinical isolate of A. baumannii ST-2 harboring the *bla*_OXA 24/40_ β-lactamase gene and *abKA/abkB* genes (toxin-antitoxin system) in a plasmid. The study of these mechanisms (bacterial tolerance and persistence) is key to the development of new anti-infective treatments which will allow for the eradication of multidrug resistant pathogens.

### Accession number(s).

The whole-genome sequence (WGS) studies of GEIH-2010 isolate Ab-2_clon_2010 comprise part of the II Spanish Multicenter Study. GEIH-REIPI A. baumannii 2000 to 2010 project (umbrella GenBank BioProject number PRJNA422585), as well as the transcriptomic results shown in GenBank BioProject number PRJNA433173 (GEO series number GSE110207). The WGSs of the A. baumannii strain ATCC 17978 complete genome and A. baumannii strain ATCC 19606 complete genome are deposited under GenBank accession numbers CP018664.1 and GG704575.1, respectively.

## Supplementary Material

Supplemental material
