# Supplementary material for: Relationship between Tolerance and Persistence Mechanisms in Acinetobacter baumannii Strains with AbkAB Toxin-Antitoxin System
Source: Antimicrob Agents Chemother. 2018 Apr 26;62(5):e00250-18. doi: 10.1128/AAC.00250-18 (PMC5923160; doi:10.1128/AAC.00250-18)
Supplement: Supplemental material [file supp_62_5_e00250-18__index.html]

Supplemental material 

# Relationship between Tolerance and Persistence Mechanisms in Acinetobacter baumannii Strains with AbkAB Toxin-Antitoxin System

## Supplemental material

- Supplemental file 1 -

  Supplemental material

  PDF, 247K
