# Supplementary material for: Relationship between Tolerance and Persistence Mechanisms in Acinetobacter baumannii Strains with AbkAB Toxin-Antitoxin System
Source: Antimicrob Agents Chemother. 2018 Apr 26;62(5):e00250-18. doi: 10.1128/AAC.00250-18 (PMC5923160; doi:10.1128/AAC.00250-18)
Supplement: Supplemental material [file AAC.00250-18_zac005187102s1.pdf]

	MIC (mg/L)														
**Isolate**	**SUL**	**CAZ**	**IMP**	**MEM**	**CIP**	**GEN**	**TOB**	**AMK**	**MIN**	**NET**	**TET**	**DOX**	**CST**	**RIF**	**TGC**
Ab-2_clon_2010	64	>128	64	32	32	2	32	32	<0.5	>64	>64	16	<0.5	1	0.5
Ab-2_clon_2010-CHLX	32	>128	64	32	32	2	32	16	<0.5	64	>64	16	<0.5	1	0.5

Table S1. MICs of several antimicrobials for the bacterial isolates used in the study.

	GO. ID	Term	Annotated	Significant	Expected	ClassicFisher
1	GO:0030001	metal ion transport	27	20	11.28	0.00063
2	GO:0006813	potassium ion transport	11	10	4.6	0.00108
3	GO:0015672	monovalent inorganic cation transport	25	18	10.45	0.00206
4	GO:0006812	cation transport	46	29	19.22	0.00266
**5**	**GO:0019222**	**regulation of metabolic process**	**213**	**105**	**89.02**	**0.01142**
**6**	**GO:0032774**	**RNA biosynthetic process**	**211**	**104**	**88.18**	**0.01187**
**7**	**GO:0060255**	**regulation of macromolecule metabolic process**	**211**	**104**	**88.18**	**0.01187**
8	GO:0006805	xenobiotic metabolic process	5	5	2.09	0.01265
9	GO:0009410	response to xenobiotic stimulus	5	5	2.09	0.01265
10	GO:0010124	Phenylacetate catabolic process	5	5	2.09	0.01265

Table S2. Using the TopGO software tool (version 3.3) and a Fisher test, the GO term enrichment analysis was performed. This enrichment has been made from the previously made InterProtScan annotation that allowed us to relate 1852 genes with GO terms, of which 774 were included within the SDRs identified in common by the software EdgeR and DESeq2. This analysis has made it possible to identify that there is an enrichment of the differentially expressed genes that are associated with the terms GO: 0030001 ("metal ion transport"), GO: 0006813 ("potassium ion transport"), GO: 0015672 ("monovalent inorganic cation" transport ") and GO: 0006812 (" cation transport "). The reference value to consider a term as significant has been p-value <0.01. In this Table S1, it shows the enrichment values according to the GO terms.

PRIMERS and PROBES for RT-PCR STUDIES				
**Genes /Function**		**Primer Sequences (5´-3´)**	**Taqman Probes**	**References**
abaR/Quorum Sensing	Fow	ACCTCTTGTTTGGTCGAGTCA	96/ACAGGCAG	(1)
	Rev	CGTGCTTCCTCCCAAAAAT		
abaI/ Quorum Sensing	Fow	CCGCTACAGGGTATTTGTTGAAT	6FAM-TGGATTCTCTGTCTTGAGCCACGACA-BBQ	(1)
	Rev	GCAGGGAATAGGCATTCCATTG		
bla$_{OXA24}$ ß-lactamase	Rev	CAAATAAAGAATATGTCCCTGC	-	This study[a]
	Fow	CTGCATTAGCTCTAGGCCAGT		
abkA antitoxin	Rev	GATAACCTTTGCCTTGTGC	-	This study[a]
	Fow	CGCTTGCTTGCTAAAGGCAC		
abkB toxin	Rev	CTCATATTTGTTTCGCTCCGC	-	This study[a]
	Fow	GATCCATTACGGATCTCAATCC		

Table S3. Primers and probes used in this study. [a]Using LightCycler FastStrand DNA Master SYBR green I (Roche, Mannheim, Germany).

REFERENCES

1. López M, Mayer C, Fernández-García L, Blasco L, Muras A, Ruiz FM, Bou G, Otero A, Tomás M; GEIH-GEMARA (SEIMC). 2017. Quorum sensing network in clinical strains of *A. baumannii*: AidA is a new quorum quenching enzyme. PLoS One. 12(3):e0174454.

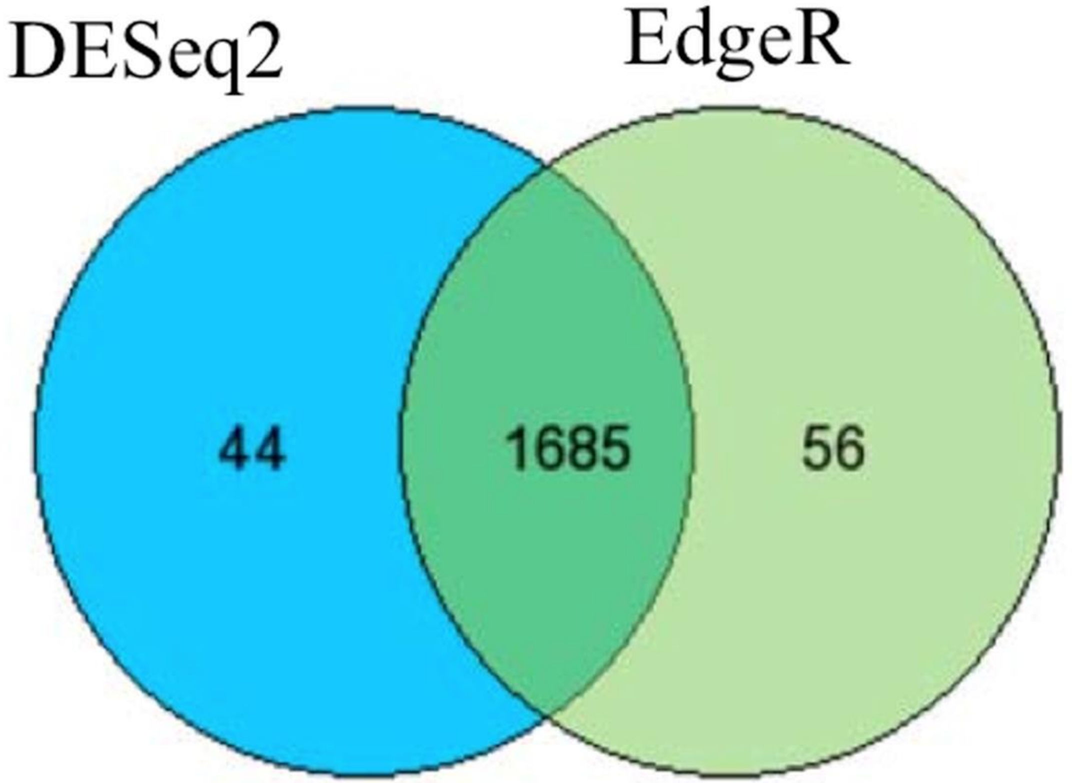

Fig 1S. Venn Diagram. Total number of genes differentially expressed between the groups (RNAs from Ab-2_clon_2010 *versus* Ab-2_clon_2010-CHLX) identified in common between both methods. With respect to the previous report, we observed a greater number of differentially expressed genes identified by both packages (1685 vs 1204 differentially expressed genes).

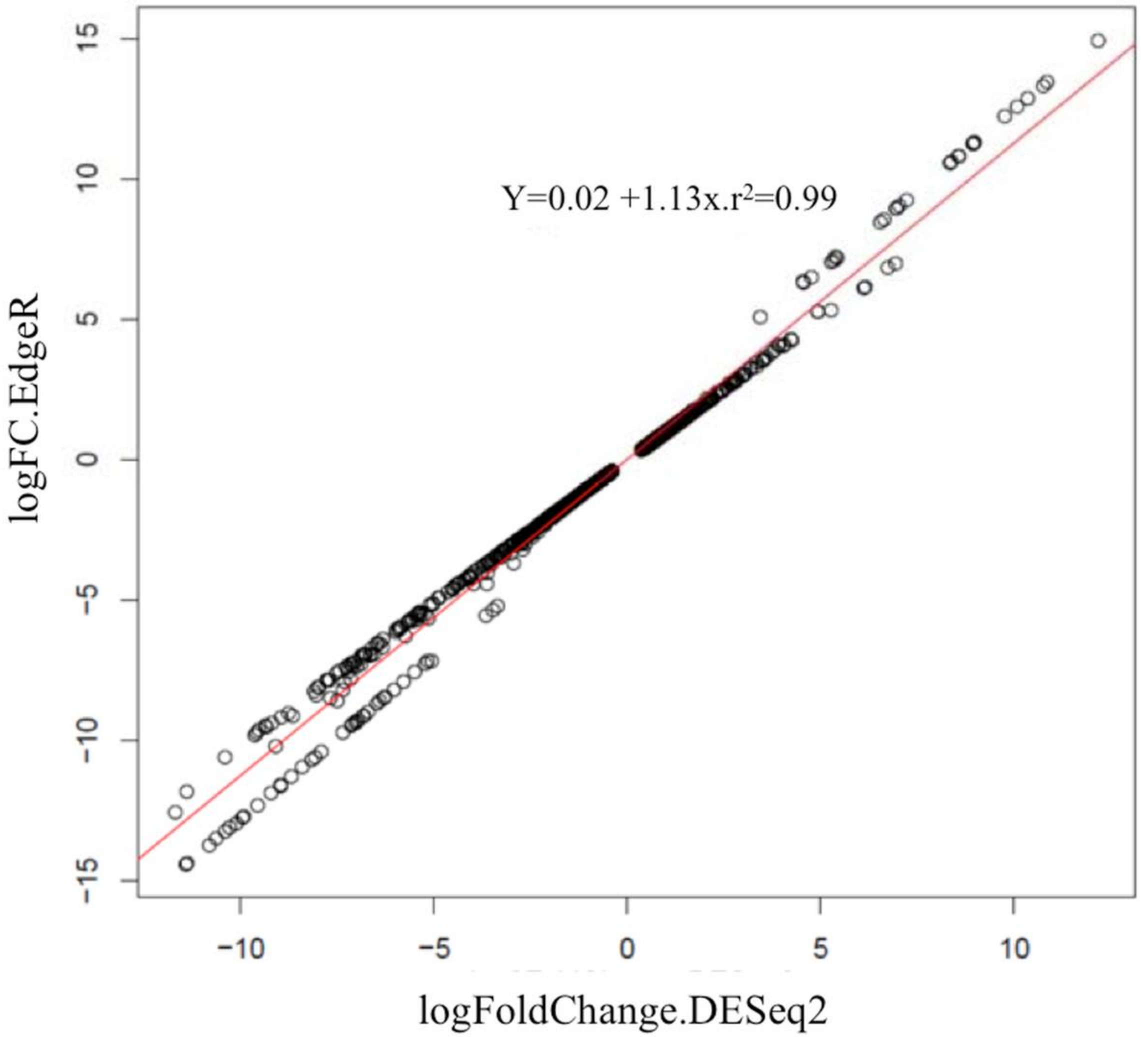

Figure 2S. Comparison of the LogFoldChange identified by EdgeR and DESeq2 for each of the differentially expressed genes.
